# Mefloquine exerts anticancer activity in prostate cancer cells via ROS-mediated modulation of Akt, ERK, JNK and AMPK signaling

**DOI:** 10.3892/ol.2013.1211

**Published:** 2013-02-22

**Authors:** KUN-HUANG YAN, CHIH-JUNG YAO, CHI-HAO HSIAO, KE-HSUN LIN, YUNG-WEI LIN, YU-CHING WEN, CHUNG-CHI LIU, MING-DE YAN, SHUANG-EN CHUANG, GI-MING LAI, LIANG-MING LEE

**Affiliations:** 1 Department of Urology, Wan Fang Hospital, Taipei Medical University, Taipei Medical University, Taipei 11696;; 2 Department of Internal Medicine, School of Medicine, College of Medicine, Taipei Medical University, Taipei 11696;; 3 Cancer Center, Wan Fang Hospital, Taipei Medical University, Taipei 11696;; 4 Center of Excellence for Cancer Research, Taipei Medical University, Taipei 11696;; 5 Division of Gastroenterology, Department of Internal Medicine, Wan Fang Hospital, Taipei Medical University, Taipei 11696;; 6 National Institutes of Cancer Research, National Health Research Institutes, Miaoli 35053;; 7 Division of Hematology and Medical Oncology, Department of Internal Medicine, Wan Fang Hospital, Taipei Medical University, Taipei 11696, Taiwan, R.O.C.; 8 Department of Urology, School of Medicine, College of Medicine, Taipei Medical University, Taipei 11696, Taiwan, R.O.C.

**Keywords:** mefloquine, prostate cancer, reactive oxygen species, mitochondrial membrane potential, hyperpolarization, N-acetyl cysteine

## Abstract

Mefloquine (MQ) is a prophylactic anti-malarial drug. Previous studies have shown that MQ induces oxidative stress *in vitro*. Evidence indicates that reactive oxygen species (ROS) may be used as a therapeutic modality to kill cancer cells. This study investigated whether MQ also inhibits prostate cancer (PCa) cell growth. We used sulforhodamine B (SRB) staining to determine cell viability. MQ has a highly selective cytotoxicity that inhibits PCa cell growth. The antitumor effect was most significant when examined using a colony formation assay. MQ also induces hyperpolarization of the mitochondrial membrane potential (MMP), as well as ROS generation. The blockade of MQ-induced anticancer effects by N-acetyl cysteine (NAC) pre-treatment confirmed the role of ROS. This indicates that the MQ-induced anticancer effects are caused primarily by increased ROS generation. Moreover, we observed that MQ-mediated ROS simultaneously downregulated Akt phosphorylation and activated extracellular signal-regulated kinase (ERK), c-Jun N-terminal kinase (JNK) and adenosine monophosphate-activated protein kinase (AMPK) signaling in PC3 cells. These findings provide insights for further anticancer therapeutic options.

## Introduction

Prostate cancer (PCa) is the most frequently diagnosed malignancy in males and a common cause of cancer-related mortality. Hormone deprivation is an option for rapid androgen ablation in PCa patients. However, cancer cells eventually recur and progress to the androgen-independent stage. The treatments available for patients during this stage are limited.

Chloroquine (CQ) is a traditional anti-malarial medication that has been identified as a potential adjuvant in the treatment regimen of glioblastoma multiforme (GBM) (1). The results of clinical trials (2,3) indicate that CQ is a potential adjuvant therapy for glioblastoma. Moreover, mefloquine (MQ) has been shown to be more potent than CQ in killing cancer cells *in vitro* and is potentially more efficacious than CQ as a chemotherapeutic agent for GBM patients (1). MQ is a valuable anti-malarial drug for prophylaxis and treatment for the majority of patients (4). MQ plasma concentrations reached 1,692 ng/ml (4.48 *μ*M) for chemosuppression in *Plasmodium falciparum* infections (5,6). Krudsood *et al*(7) reported that an MQ plasma concentration of 5,796 ng/ml (15.35 *μ*M) was attained in a clinical study on *P. falciparum*-infected adults. Children tolerate MQ better than adults, and males tolerate it better than females (8).

This study examined two PCa cell lines (DU145 and PC3). DU145 and PC3 are the most commonly used PCa cell lines and their characteristics differ. PC3 was isolated from a bone metastasis, whereas DU145 cells were isolated from the brain metastases of prostate carcinoma. However, p53-mutant DU145 (9,10) and p53-null PC3 (9,11) cells are androgen-independent and proliferate normally in androgen-deprived media.

This study had the following two objectives: i) to determine whether MQ possesses anticancer effects at potential therapeutic concentrations *in vitro*; and ii) to detail general MQ features in the context of disrupted cancer cell proliferation. This study shows that MQ exposure causes immediate hyperpolarization of mitochondrial membrane potential (MMP) and increases reactive oxygen species (ROS) generation in PCa cells. For the first time, the study also shows that MQ-mediated ROS inhibits Akt phosphorylation and activates c-Jun N-terminal kinase (JNK), extracellular signal-regulated kinase (ERK) and adenosine monophosphate-activated protein kinase (AMPK) signaling.

## Materials and methods

### Cell culture

Human foreskin fibroblast Hs68 cells and the androgen-independent PCa cell lines, PC3 and DU145, were maintained in Dulbecco’s modified Eagle’s medium and supplemented with 10% fetal bovine serum. The PCa cells were continuously cultured in a regular cell culture medium with 2 mM L-glutamine, 100 *μ*g/ml streptomycin and 100 U/ml penicillin in a humidified 5% CO_2_ atmosphere. This study was approved by the Taipei Medical University Wan Fang Hospital.

### Cell viability assay

Cells were seeded onto 96-well plates at a density of 5,000 cells per well and incubated for 1 day. Cell viability was assayed using sulforhodamine B (SRB) staining, as described previously (12). Absorbance at 570 nm was measured using an ELISA reader. Cell viability is expressed as the percentage of absorbance of the treated cells relative to that of the untreated (control) cells.

### Colony formation assay

One hundred cells were seeded in a 10-cm culture dish for 24 h and then incubated with MQ (or without for control). Colonies >0.5 mm were counted after 2 weeks. Colonies were washed with phosphate-buffered saline (PBS) then air-dried, stained with 0.4% crystal violet for 1 min, rinsed in water, air-dried and then photographed.

### MMP assessment

MMP was measured using 40 *μ*M cationic lipophilic fluorochrome DiOC6. Cells were treated with MQ (5×10^5^ cells/well) in a 6-well plate for 40 min, then DiOC6 was added and the cells were cultured continuously without light for 20 min at 37°C in the presence of MQ. Cells were then obtained and washed with 1 ml ice-cold PBS. Finally, the cells were suspended in PBS and analyzed immediately using flow cytometry (FC500, Beckman Coulter, Miami, FL, USA). DiOC6 was recorded by fluorescence. The mean value of the MMP in the treated cells was calculated for comparison with that of the control cells.

### Intracellular ROS assays

A 5-*μ*M non-fluorescent 2′,7′-dichlorofluorescein-diacetate (DCFH-DA) intracellular probe was used to detect ROS formation. Cells were treated with MQ (5×10^5^ cells/well) in a 6-well plate for 40 min, then DCFH-DA was added and the cells were cultured continuously without light for 20 min at 37°C. Finally, cells were collected by centrifuging and suspended in 1 ml ice-cold PBS. The oxidation of DCFH by ROS was determined by measuring the mean fluorescent intensity of DCFH by flow cytometry (FC500).

### Western blot analysis

PC3 cells were pre-treated with 10 mM NAC for 20 min and then treated with 10 *μ*M MQ for 1 h. These were compared to the control cells which were treated with 10 *μ*M MQ for 1 h only. At harvest, total protein extracts were prepared and the protein concentration was determined using the Bradford method. Aliquots containing 20 *μ*g total protein each were subjected to western blot analysis. Antibodies against glyceraldehyde-3-phosphate dehydrogenase were purchased from Santa Cruz Biotechnology (Santa Cruz, CA, USA). Antibodies against phosphorylated Akt, JNK, AMPK and ERK were purchased from Cell Signaling Technology (Danvers, MA, USA).

### Statistical analysis

Error bars represent the standard error of means (SEM) from independent triplicates (n=3). All data are expressed as mean ± SEM. We employed Sigma Plot 2001 software for statistical analysis. P<0.05 was considered to indicate a statistically significant result.

## Results

### Effects of MQ on the proliferation of PCa and Hs68 cells

**Figure 1:**
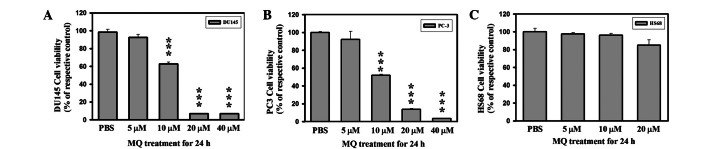
Inhibition of the proliferation of prostate cancer (PCa) cells by mefloquine (MQ) *in vitro*. (A) DU145, (B) PC3 and (C) Hs68 cells were treated with the indicated concentrations of MQ or phosphate-buffered saline (PBS; control) for 24 h, and then assayed using sulforhodamine B (SRB) staining. Relative cell viability (% of PBS control) is expressed as the mean ± standard error of the mean (SEM). Error bars show the SEM (n=3). ^***^P<0.001.

Two established PCa cell lines (DU145 and PC3) were examined for their sensitivity to MQ *in vitro.* We examined the growth inhibitory effects of MQ on PCa and Hs68 cells using SRB staining (Fig. 1). The PCa cell population was reduced after 24 h exposure to MQ (IC_50_ ∼10 *μ*M MQ, Fig. 1A and B). PCa cell proliferation was completely inhibited at MQ concentrations >20 *μ*M. Assessment of Hs68 cells following MQ treatment showed no reduction in cell viability at 10 *μ*M (Fig. 1C). Treatment of the Hs68 cells with MQ resulted in an effect of <IC_10_ at ∼20 *μ*M MQ at 24 h. Hs68 cells exhibited greater resistance to MQ exposure compared with the PCa cells. The results show that the IC_50_ value of MQ for PCa cells was ∼10 *μ*M. MQ is a highly cytotoxic drug for PCa cell lines and causes ∼50% cell death in DU145 and PC3 at clinically achievable concentrations. Despite its anticancer potency, MQ is relatively nontoxic towards normal human cells.

### Effects of MQ on colony formation in PCa cells

**Figure 2:**
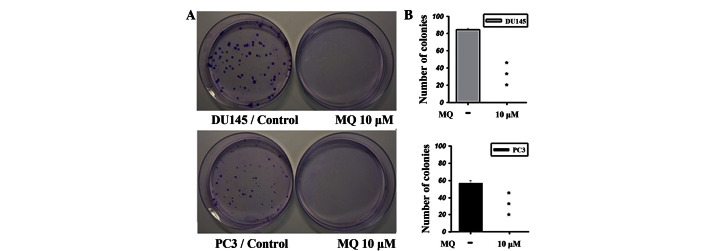
Antitumor effect of mefloquine (MQ) in DU145 and PC3 cells was assayed by colony formation assay. (A) Colony images and (B) relative number of colonies. Error bars show the standard error of the mean (SEM; n=3). ^***^P<0.001 vs. the control.

A colony formation assay was performed to further show the antitumor effect of MQ (Fig. 2). Images of colonies grown in the presence or absence of MQ are shown in Fig. 2A (top, DU145; bottom, PC3). A significant antitumor effect was observed for MQ against the DU145 and PC3 cells (Fig. 2B).

### Effects of MQ on MMP and ROS in PCa cells

**Figure 3:**
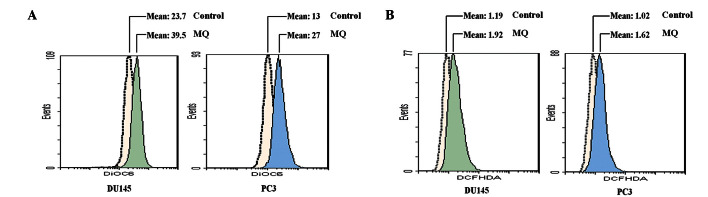
Effect of mefloquine (MQ)-induced alteration of mitochondrial membrane potential (MMP) and reactive oxygen species (ROS) generation in PCa cells. (A) DU145 and PC3 cells were treated with MQ (10 *μ*M) for 60 min, and the change in MMP was analyzed by flow cytometry with DiOC6. (B) DU145 and PC3 cells were treated with the indicated concentration of MQ (10 *μ*M) for 1 h, and the ROS level was determined using a 2′,7′-dichlorofluorescein-diacetate (DCFH-DA) dye.

MMP represents the performance of the electron transport chain and may indicate a pathological disorder of that system. A failure of mitochondrial bioenergetics is closely associated with the onset of apoptosis and necrosis. Since MQ is closely related to the alteration of MMP (13), this study investigated the effects of MQ on MMP using DiOC6. Following 1 h exposure to MQ (10 *μ*M), the fluorescence mean value of the MMP was ∼1.5- to 2-fold higher than that of the control PCa cells (Fig. 3A). Since MQ has a rapid effect on the hyperpolarization of MMP, the level of ROS produced within 1 h was further analyzed. The intracellular ROS levels in the MQ-treated PCa cells were assessed (Fig. 3B). PCa cells have a lower mean DCFH value in the absence of MQ than in its presence. MQ significantly stimulated the generation of ROS by PCa cells, as observed by the alteration of DCFH fluorescence. When treated with 10 *μ*M MQ, the DCFH fluorescence increased from 1.19 to 1.92 and from 1.02 to 1.62 in DU145 and PC3 cells, respectively.

### Pre-treatment with N-acetyl cysteine (NAC) protects DU145 and PC3 cells against MQ-induced anticancer effects and ROS-mediated signaling

**Figure 4:**
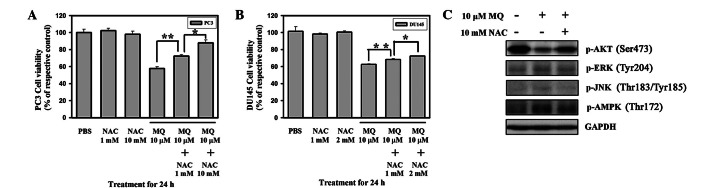
Pre-treatment with N-acetyl cysteine (NAC) disrupts mefloquine (MQ)-induced anticancer effects through reactive oxygen species (ROS) scavenging. (A) PC3 and (B) DU145 cells were pre-treated with the indicated NAC concentrations for 20 min before incubation with MQ (10 *μ*M) for 24 h and subsequent monitoring of cell viability. Relative cell viability [% of phosphate-buffered saline (PBS) control] is expressed as the mean ± standard error of the mean (SEM). Error bars show SEM (n=3). ^*^P<0.05; ^**^P<0.01. (C) MQ-mediated signal transduction involving ROS-induced signaling alteration was also assayed. PC3 cells were seeded in 6-well plates for 24 h and then drug-treated for 1 h. Cell lysates were analyzed by western blot analysis for p-Akt, p-JNK, p-ERK and p-AMPK. JNK, c-Jun N-terminal kinase; ERK, extracellular signal-regulated kinase; AMPK, adenosine monophosphate-activated protein kinase.

Previous studies have suggested that preincubation with NAC is required to inhibit the cytotoxicity of generated ROS (14–16). NAC, which contains a sulfhydryl group, acts as a ROS scavenger and a precursor of intracellular reduced glutathione to regulate the redox status in the cells. To confirm the role of ROS in MQ-induced anticancer effects, cell viability was detected in DU145 and PC3 cells pre-treated with NAC for 20 min and then treated with 10 *μ*M MQ for 24 h. Cell viability was determined using SRB staining. NAC pre-treatment significantly inhibited MQ-induced anticancer effects in PC3 and DU145 cells (Fig. 4A and B). The NAC control groups (PC3, 1 and 10 mM; DU145, 1 and 2 mM) showed no significant changes in cell viability compared with the untreated controls. These results indicate that MQ-mediated cytotoxicity is quenched by the antioxidant NAC. These data may also indicate that increased ROS generation is essential in MQ-mediated cell death. MQ-mediated signal transduction pathways involving ROS-induced signaling modulation were also investigated (Fig. 4C). We observed that MQ-mediated ROS simultaneously downregulated Akt Ser473 phosphorylation and activated ERK, JNK and AMPK signaling in PC3 cells. Moreover, Akt signaling was rescued and phosphorylation of ERK Tyr204, JNK Thr183/Tyr185 and AMPK Thr172 was decreased by pre-treatment with NAC, the ROS scavenger.

## Discussion

MQ is a prophylactic anti-malarial drug that has been shown to possess antineoplastic activity against an experimental GBM tumor (1). Dow *et al*(17) showed that higher MQ blood levels are achieved under therapeutic regimens (2.1 to 23 *μ*M) than under prophylaxis (3.8 *μ*M). This study was designed to understand the efficacy underlying MQ-induced anticancer effects. Human PCa cell lines, including commonly used androgen-independent DU145 and PC3 (18), were used.

Exposure to 10 *μ*M MQ for 24 h decreased the populations of DU145 and PC3 cells as determined by SRB staining (Fig. 1). The clonogenic effect was most significantly diminished when examined using a colony formation assay (Fig. 2). However, a normal human skin fibroblast cell line (Hs68) exhibited a different response to MQ exposure. Hs68 cells continued to proliferate normally in the presence of 10–20 *μ*M MQ, whereas the cancer cells were sensitive to the cytotoxic effects of MQ at 10 *μ*M. This shows that MQ has highly selective anticancer capabilities.

Mitochondria are required as the primary source of ROS in cells. Mitochondrial dysfunction, oxidative stress and impaired cerebral energy metabolism contribute to cell death (19). The mitochondria are the primary organelles for production of high-energy phosphate. MMP hyperpolarization has also been associated with subcellular necrosis (20). In cell suicide (apoptosis and necrosis), mitochondrial hyperpolarization is required for specific steps of cell death (21). Previous studies have indicated that MQ treatment induces the cytotoxic effect that is dependent on the increase in oxidative stress in primary rat cortical neurons (22). MMP hyperpolarization may also increase ROS production (23). Exogenous oxidative stress may induce transient hyperpolarization of MMP and a delayed burst of endogenous ROS (24). MMP hyperpolarization and ROS generation are early molecular events that precede cell death (25). In certain types of necrosis, cell death is mediated by an increase in the MMP-dependent overproduction of ROS (21). A recent study (26) has shown that massive mitoptosis may result in cell death. ROS-initiated mitoptosis is presumed to eliminate the mitochondria which overproduce ROS (21). MMP hyperpolarization-induced ROS cause oxidative stress-induced necrotic cell death (24).

MMP alteration and oxidative stress in cancer cells treated with MQ have not been examined previously. The current study investigated MMP alteration induced by 1 h of exposure to MQ in PCa cells. In the alteration of MMP, a change in the state of ROS was also observed. In our study, MQ treatment led to significant MMP hyperpolarization. This indicates that MMP hyperpolarization during MQ treatment mediated intracellular ROS generation which inhibited PCa cells at concentrations >10 *μ*M.

There are several signaling factors, such as Akt, AMPK and the mitogen-activated protein kinase (MAPK) family, that regulate the progression of various tumors. Akt is activated by Ser473 phosphorylation, which affects cell growth, proliferation and survival. The increased ROS levels may enhance MAPK activities. The phosphorylation of AMPK Thr172 depends on cellular metabolic stress. Previous studies have implied that the signaling factors Akt, AMPK and the MAPK family are potentially associated with ROS-triggered biological responses. ROS are a significant type of molecule that mediate numerous signal transduction pathways and have a critical function in cells. To confirm the function of ROS generation in MQ treatment, NAC was used to scavenge the ROS. Pre-treatment of DU145 and PC3 cells with NAC abrogated ROS induction by MQ and significantly protected these cells against MQ-induced anticancer effects. Exposure of PC3 cells to MQ for 1 h caused a decrease in phosphorylation of Akt at Ser473. Simultaneously, we also observed the activation of AMPK (at Thr172) and MAPK family members (ERK at Tyr204 and JNK at Thr183/Tyr185) in response to MQ treatment. Pre-treatment with NAC confirmed the function of Akt, ERK, JNK and AMPK in MQ treatment. Blocking ROS generation with NAC had a significantly preventative effect on Akt activation, and inhibited ERK, JNK and AMPK. This study indicates an anticancer action of MQ that is dependent on Akt signaling disruption and JNK, ERK and AMPK signaling upregulation in PCa cells; this action is dependent on ROS generation. Mitochondrial function and ROS generation are involved in PI3K-Akt-mTOR and MAPK (JNK/ERK) signaling for autophagy in cancer (27). AMPK regulates the ROS/redox balance which indicates that AMPK signaling is critical in intracellular homoeostasis during autophagy (28). The intricate relationships between ROS-mediated signaling (involving Akt, JNK, ERK and AMPK) and autophagy require further study. We corroborate that MQ induces ROS generation which then affects cancer cell proliferation by modulating signaling transduction, specifically by ERK, JNK and AMPK activation and Akt inhibition.

Thus, we assert that MQ has a strong inhibitory effect on cell viability by producing ROS in DU145 and PC3 cells. These results indicate that MQ may be a potential candidate for clinical trials of cancer applications in the future.

## Abbreviations:

MQ: mefloquine;
PCa: prostate cancer;
ROS: reactive oxygen species;
MMP: mitochondrial membrane potential;
NAC: N-acetyl cysteine
